# Comparing dietary macronutrient composition and food sources between native and diasporic Ghanaian adults

**DOI:** 10.3402/fnr.v59.27790

**Published:** 2015-11-24

**Authors:** Rachel Gibson, Annemarie Knight, Matilda Asante, Jane Thomas, Louise M. Goff

**Affiliations:** 1 Department of Nutrition and Dietetics, King's College London, London, England; 2 Department of Nutrition and Dietetics, School of Allied Health Sciences, College of Health Sciences, University of Ghana, Korle-Bu Accra, Ghana

**Keywords:** West African, diet, ethnicity, nutrition

## Abstract

**Background:**

Dietary acculturation may contribute to the increased burden of non-communicable diseases (NCDs) in diasporic populations of African ancestry.

**Objective:**

To assess nutritional composition and the contribution that traditional foods make to the diets of native and UK-dwelling Ghanaian adults.

**Design:**

An observational study of Ghanaian adults living in Accra (*n*=26) and London (*n*=57) was undertaken. Three-day food records were translated to nutrient data using culturally sensitive methods and comparisons were made for energy, macronutrients, and dietary fibre between cohorts. The contribution of traditional foods to dietary intake was measured and the foods contributing to each nutrient were identified.

**Results:**

Compared to native Ghanaians, UK-Ghanaians derived a significantly higher proportion of energy from protein (16.9±3.9 vs. 14.1±2.8%, *p=*0.001), fat (29.9±7.9 vs. 24.4±8.5%, *p*=0.005), and saturated fat (8.5±3.4 vs. 5.8±3.7%, *p*<0.001) and a significantly lower energy from carbohydrate (52.2±7.7 vs. 61.5±9.3%, *p<*0.001). Dietary fibre intake was significantly higher in the UK-Ghanaian diet compared to the native Ghanaian diet (8.3±3.1 vs. 6.7±2.2 g/1,000 kcal, *p*=0.007). There was significantly less energy, macronutrients, and fibre derived from traditional foods post-migration. Non-traditional foods including breakfast cereals, wholemeal bread, and processed meats made a greater contribution to nutrient intake post-migration.

**Conclusions:**

Our findings show the migrant Ghanaian diet is characterised by significantly higher intakes of fat, saturated fat, and protein and significantly lower intakes of carbohydrate; a macronutrient profile which may promote increased risk of NCDs amongst UK-Ghanaians. These differences in the nutrient profile are likely to be modulated by the consumption of ‘Western’ foods observed in migrant communities.

Diet-related ill health places a large burden on global economies. In the United Kingdom, the annual healthcare costs of diet-related conditions such as obesity, hypertension, and type 2 diabetes are estimated at £5.8 billion (1). All of these diseases are more prevalent amongst those of Black African ethnicity residing in the United Kingdom compared to the general UK population (2). Against the backdrop of an increasingly diverse ethnic population, inequalities in UK healthcare provision are evident despite numerous government initiatives (3). Lifestyle interventions form the cornerstone of management of these chronic conditions therefore nutritional practitioner knowledge of cultural dietary patterns plays an important part in understanding disease risk and in the development of culturally appropriate nutritional interventions (4). Culturally tailored healthcare, that is respectful of and responsive to the health beliefs, practices, and cultural and linguistic needs of diverse patients, has been shown to bring about positive health outcomes (5).

West Africans, mainly migrants from Ghana and Nigeria, account for the largest proportion of those classified as Black African in the UK population (6). To date, nutritional research in West African populations has been focused on dietary pattern analysis, recognising the impact of the nutrition transition in West African countries (7–10). While migration studies have demonstrated the increased prevalence of a number of diet-related non-communicable diseases (NCDs) including colorectal cancer (11), dysglycaemia (12), and obesity (13, 14) amongst diasporic West African origin populations, there has been very limited research describing the diet and changes in diet that occur in migratory communities of West African origin. The aim of the present work was to assess the dietary intake of a cohort of UK-dwelling and native adult Ghanaians. Specifically, we aimed to compare the nutritional composition of the diet, the main food sources, and the contribution that traditional foods make to dietary intake between native and migrant Ghanaian adults.

## Methods

An exploratory observational study was conducted to characterise the dietary intake of UK-residing first-generation Ghanaians compared to Ghanaians residing in their native country.

### Study population

Healthy adult male and female participants were recruited through contact with Ghanaian community groups in Ghana and London. Participants were eligible to participate if they were born in Ghana, >18 years, and not pregnant, lactating, diabetic, or with any major chronic disease, and not following a special diet. This study was conducted according to the guidelines laid down in the Declaration of Helsinki and all procedures involving human subjects were approved by the Research Ethics Committees of King's College London and University of Ghana Medical School.

### Procedures

Dietary intake data were collected using 3-day food records (2 weekdays and 1 day at the weekend). Participants were asked to indicate their portion sizes using a photographic atlas, ethnically appropriate photographs and Ghanaian household measures (e.g. ladles to describe soups and stews) (15). Anthropometric data were collected using a portable stadiometer to record height to the nearest 0.1 cm and weighing scales (Seca 762, Vogel and Halke, Germany) to measure weight to the nearest 0.1 kg (including light clothing). Body mass index (BMI) was calculated as weight (kg)/height (m^2^). Additionally, participants completed a short socio-demographic questionnaire that provided details on their age, sex, and employment status (length of UK residency was additionally included in the UK questionnaire).

### Dietary data analysis

Nutritional assessment of the dietary records was performed using Dietplan6 nutritional software (Forestfield Software Ltd., Horsham, UK), which is based on The McCance and Widdowson's Composition of Foods Integrated Dataset. As part of an on-going study of traditional West African foods and dishes in our group, an extensive database of recipe and direct chemical analysis of composite dishes was utilised in the nutritional analysis. Additional sources of nutrient data included manufacturer nutrient declarations and The West African Food Table (16). A systematic approach was developed to enable robust translation of recorded foods into portion weights (grams). Resources consulted included photographic aids (17, 18), published UK portion data (19, 20), and published research relating to ethnic portion sizes (21).

Mean intakes for energy, total fat, saturated fat, carbohydrate, non-starch polysaccharides (NSP), and protein were calculated for each subject, macronutrient intakes are expressed as a percentage of total energy intake. The percentage energy contribution per macronutrient and NSP per 1,000 kcals was used to compare mixed gender cohorts to account for differences between male and female energy intakes. The percentage contribution that specific food categories made to each nutrient for each cohort was calculated by exporting the individual participant nutritional assessments from Dietplan6 to Excel (Microsoft for Mac, version 14.2.3) and assigning category codes for similar foods. Ranking the percentage contributions from each category determined the ‘top 10’ contributing food categories for each macronutrient and NSP. Calculation of the amount of traditional Ghanaian foods (TGF) that contribute to macronutrient and NSP intake per participant was based on applying the European Union definition of a traditional food: ‘‘proven usage in the community market for a time period showing transmission between generations; this time period should be the one generally ascribed as one human generation, at least 25 years’’ (22).

To apply this definition to the food lists, a consensus opinion of four registered dietitians was sought. The dietitians were representative of this ethnic group or highly experienced in working with this ethnic group.

To calculate the potential prevalence of under-reporting, basal metabolic rate was estimated using the Schofield equation (23), and a physical activity level of 1.2 was applied as the lower cut-off for under-reporting (24).

### Statistical analysis

Statistical analyses were performed using IBM SPSS Statistics version 21 (SPSS Inc., Chicago, IL). All continuous data were tested for normality using Shapiro–Wilk test (*p>*0.05 signified normal distribution). If data was not normally distributed, it was transformed where possible to allow for parametric testing. The method of transformation is stated where used. To compare parametric data between the two groups, independent *t-*tests were conducted and non-parametric data were compared using Mann–Whitney *U* test. Data are mean±standard deviation, unless otherwise indicated. Categorical data differences were compared via chi-squared test. Statistical significance is reported as *p<*0.05.

## Results

### Socio-demographic and anthropometric comparison between cohorts

A total of 83 participants were included in the analysis: 26 in the Ghana cohort, 57 in the UK cohort. Socio-demographic characteristics of each cohort are presented in Table 1. The Ghana cohort comprised 61% male; mean age 38.6±8.4 years. The UK cohort comprised 51% male; mean age 41.3±11.5 years. There were no significant differences reported for age between the two cohorts (*p=*0.305). For the UK cohort, the median length of UK residence was 11.0 years (IQR 8.0–23.2) and the mean age at migration was 26.7±8.0 years. There was no significant difference between men and women for length of UK residence or age at migration within the UK cohort. More than 60% of men and women within each cohort were in full-time employment. Height and weight data was obtained for 70% of the Ghana cohort and 70% of the UK cohort. There was no significant difference between the two cohorts for weight (*p=*0.602) or BMI (*p=*0.545) (Table 1).

**Table 1:** Socio-demographic characteristics of Ghanaian participants residing in Ghana and the United Kingdom

	Ghana			UK			
			
*n*	Men 16	Women 10	Combined 26	Men 29	Women 28	Combined 57	*p*
Age (years)							
Mean	39.9	36.8	38.6	41.9	40.6	41.3	0.305^a^
SD	7.3	9.9	8.4	10.5	11.0	11.5	
Length of residence in the UK (years)							
Median	–	–		11.0	11.5	11.0	0.962^b^
IQR	–	–		8.0–24.0	7.25–21.5	8.0–23.2	
Age at migration (years)							
Mean	–	–		27.4	26.1	26.7	0.547^a^
SD	–	–		7.5	8.59	8.0	
Weight (kg)							
Median	76.5	69.5	71.5	79.9	72.8	76.2	0.602^c^
IQR	69.3–83.25	69.5–80.5	66.3–81.0	66.5–84.7	67.65–80.25	67.2–84.4	
BMI (kg/m^2^)							
Median	27.7	25.7	27.4	27.8	26.4	26.7	0.545^c^
IQR	26.4–29.5	23.4–27.9	23.8–29.0	23.9–29.1	24.3–29.5	24.2–29.2	
Employment status (%)							
Employed	69	60	65	69	89	79	0.410^d^
Unemployed	0	0	0	0	0	0	0.141^d^
Not stated	25	20	23	0	0	0	
Student	6	20	12	17	7	12	0.510^d^

a Independent *t*-test (two-tailed) compared mean age between the two cohorts and mean age at migration between men and women within the London cohort.

b Mann–Whitney *U* test compared median length of UK residence within the London cohort.

c Mann–Whitney *U* test compared median weight and BMI between mixed-gender Accra and London Cohorts.

d Chi-squared test compared differences in employment status between mixed-gender Accra and London Cohorts.

### Differences in nutrient composition between cohorts

Table 2 details the comparisons made between the two cohorts for intakes of energy, macronutrients, and NSP. There were significant differences between the Ghana and the UK cohort for energy intake, all components of the macronutrient profile, and NSP concentration. Mean energy from saturated fat was higher in the UK cohort compared to the Ghana cohort (8.5±3.4% vs. 5.8±3.7%, *p<*0.001). Dietary fibre concentration was also significantly higher in the UK cohort when compared to the Ghana cohort (8.3±3.1 vs. 6.7±2.2%, *p=*0.007). The percentage of energy derived from carbohydrate was significantly lower in the UK cohort compared to the Ghana cohort (52.2±7.7% vs. 61.5±9.3%, *p<*0.001). The estimated prevalence of potential under-reporting, defined as reported energy intake less than BMR×1.2, was significantly higher in the UK cohort compared to the Ghana cohort (62% in the UK cohort vs. 28% in the Ghana cohort; *χ* ^2^=5.99, *p*=0.014).

**Table 2:** The contribution of energy, protein, carbohydrate, fat, saturated fat, and non-starch polysaccharide (NSP) to daily intake and, the contribution of traditional Ghanaian foods to the intake of these nutrients by location of residency

	Ghana (*n*=26)				UK (*n*=57)					
				
	Daily intake		% contribution to intake by traditional Ghanaian foods		Daily intake		% contribution to intake by traditional Ghanaian foods			
				
	Mean	SD	Median	IQR	Mean	SD	Median	IQR	*p* a	*p* b
Energy (kcal/day)	2,460	779	96.5	94.8–97.6	1,853	548	69.5	55.7–80.5	<0.001^c^	<0.001
Protein (%)	14.1	2.8	98.0	96.4–95.9	16.9	3.9	79.8	65.0–86.3	0.001^c^	<0.001
Carbohydrate (%)	61.5	9.3	96.2	93.2–98.6	52.2	7.7	69.2	52.4–77.0	<0.001	<0.001
Fat (%)	24.4	8.5	96.2	91.1–100	29.9	7.9	71.6	58.3–84.9	0.005	<0.001
Saturated fat (%)	5.8	3.7	93.3	84.3–100	8.5	3.4	65.4	54.2–82.2	<0.001^c^	<0.001
NSP (g per 1,000 kcal)	6.7	2.2	100.0	96.7–100	8.3	3.1	59.8	49.5–80.0	0.007	<0.001

SD, standard deviation; IQR, interquartile range; NSP, non-starch polysaccharides values based on Englyst measurement procedure; TEI, total energy intake. Macronutrient intakes expressed as percentage (%) of total energy intake.

a Differences in nutrient intakes between Ghana and UK groups, tested using independent samples *t*-test.

b Differences in contribution of traditional foods between Ghana and UK groups, tested using Mann–Whitney *U* tests to compare median values.

c Data transformed by square root to enable comparison of the means by independent samples *t*-test.

### TGF contribution to nutrient intakes

TGF accounted for 96.5% (IQR 94.8–97.6) of energy intake in the Ghana cohort, compared to 69.5% (IQR 55.7–80.5; *p<*0.001) in the UK cohort. The percentage of energy, macronutrients, and NSP derived from TGF are displayed in Table 2 and shows a significant difference in the contribution that TGF made to nutrient intakes between the two cohorts. All of the dietary fibre in the Ghana diet was obtained from TGF sources compared to 59.8% (IQR 49.5–80.0) in the UK-based diet. Protein was the macronutrient with the highest median contribution from TGF (79.8%, IQR 65.0–86.3) in the UK diet, while fibre had the lowest median contribution from TGF (59.8%, IQR 49.5–80.0).

### Differences in the foods that contribute to nutrient intake across cohorts

The principal 10 foods contributing to each nutrient for each cohort are listed in Tables 3 and 4. The importance of traditional cultural foods can be seen in both cohorts, for example Ghanaian soups and stews were ranked first for their contribution to fat, saturated fat, and NSP across both cohorts; they were also a major contributor to energy intake for both cohorts (Ghana 14%, UK 15%). In the UK cohort, confectionery was the fourth highest contributor to energy intake but did not feature in the top 10 energy foods in the Ghana cohort; similarly other Western food groups such as breakfast cereals featured in the UK top 10 energy contributors but did not feature in the Ghana list. Processed meat contributed to 4% of fat and 5% of saturated fat intake for the UK cohort; this product category did not feature in the top 10 foods contributing to any nutrient in the Ghana cohort. In the Ghana cohort, 46% of protein intake was derived from soups and stews, and fish compared to 35% in the UK cohort; poultry was a more important source of protein for the UK cohort than the Ghana cohort, accounting for 11% compared to 2% of protein intake, respectively. Breakfast cereals and wholemeal bread featured in the top 10 foods for energy, carbohydrate, and NSP in the UK cohort but did not feature in the Ghana cohort. Potato and potato products ranked seventh in their contribution to NSP in the UK cohort, but no participant reported consuming these foods in the Ghana cohort. Cow hide and coconut contributed to fat and saturated fat in the Ghana cohort, neither of these foods were recorded by the UK cohort.

**Table 3:** Top 10 foods contributing to total energy, protein, and carbohydrate intake (represented as % of intake), by location of residency

	Energy intake				Protein intake				Carbohydrate intake			
			
	Ghana		UK		Ghana		UK		Ghana		UK	
						
Rank	Food items	%	Food items	%	Food items	%	Food items	%	Food items	%	Food items	%
1	Fufu	16	Soups and stews	15	Fish	23	Soups and stews	23	Fufu	26	White rice	12
2	Soups and stews	14	White rice	7	Soups and stews	23	Fish	12	Kenkey	16	Fufu	11
3	Kenkey	12	Fufu	6	Kenkey	10	Poultry	11	Banku	7	Kenkey	7
4	Banku	5	Confectionery	5	Red meat (unprocessed)	8	Red meat (unprocessed)	6	White rice	6	Confectionery	7
5	Plantain	5	Kenkey	5	White bread	4	Eggs	4	Plantain	6	Breakfast cereal	5
6	White rice	4	Fish	4	Rice and beans/waakye	4	Milk	4	Yam	6	Fruit	5
7	Fish	4	Poultry	3	White rice	3	Processed meat	4	White bread	5	White bread	5
8	White bread	4	Wholemeal bread	3	Starchy porridges	2	White rice	3	Starchy porridges	5	Wholemeal bread	5
9	Starchy porridges	4	Breakfast cereal	3	Cow hide	2	Kenkey	3	Rice and beans/waakye	4	Soups and stews	5
10	Yam	4	White bread	3	Poultry	2	White bread	2	Fruit	4	Yam	5
Total		72		54		81		72		85		67

**Table 4:** Top 10 foods contributing to fat, saturated fat, and non-starch polysaccharide intake (represented as % of intake), by location of residency

	Total fat intake				Saturated fat intake				Non-starch polysaccharide intake			
			
	Ghana		UK		Ghana		UK		Ghana		UK	
						
Rank	Food items	%	Food items	%	Food items	%	Food items	%	Food items	%	Food items	%
1	Soups and stews	35	Soups and stews	27	Soups and stews	35	Soups and stews	27	Soups and stews	33	Soups and stews	24
2	Shito	7	Jollof rice	6	Coconut	10	Confectionery	8	Fruit	10	Wholemeal bread	9
3	Red meat (unprocessed)	6	Confectionery	5	Red meat (unprocessed)	10	Milk	8	Fufu	8	Fruit	9
4	Plantain	5	Eggs	5	Confectionery	5	Dairy (exc. milk)	6	White bread	7	Vegetables	8
5	Fish	5	Poultry	5	Cow hide	5	Eggs	6	Plantain	7	Breakfast cereal	8
6	Kenkey	5	Peanuts	5	Milk	4	Evaporated milk	5	Yam	6	Fufu	8
7	Cow hide	4	Fish	4	Dairy (exc. milk)	4	Processed meat	5	Vegetables	5	Potato and products	4
8	Peanuts	3	Processed meat	4	Fish	3	Red meat (unprocessed)	4	Shito	4	Yam	4
9	Coconut	3	Milk	4	Starchy porridges	3	Poultry	4	Rice and beans/waakye	4	White bread	3
10	Confectionery	3	Dairy (exc. Milk)	3	Peanuts Eggs	3	Fish	3	Coconut	4	Starchy porridges	3
Total		76		68		82		76		88		80

Soups and stews: traditional method of ‘one-pot’ cooking using a vegetable base (typically tomato, onion and pepper) with peanuts/meat/fish added. Waakye: rice cooked with bitter leaves. Fufu: starchy boiled dumpling made with cassava, yam, or plantain. Kenkey: starchy steamed dumpling made from fermented and unfermented corn. Shito: condiment added to meat or fish. Banku: starchy dumpling made with fermented corn. Confectionery: cakes, chocolate bars, sugar-sweetened beverages, biscuits, boiled sweets, jellies, and preserves.

## Discussion

In this study, we have collected detailed dietary intake data to compare the nutritional composition of the diets of first-generation diasporic Ghanaian adults residing in the United Kingdom and adult Ghanaians residing in their native country and described, for the first time, in detail important food sources in both the native and migrant diets. Our results show that the native Ghanaian diet has a lower fat, saturated, fat and protein content and relies heavily on TGFs and dishes compared to the migrant diet, which relies more heavily on processed ‘Western’ foods and is characterised by higher fat and saturated fat intakes; a nutrient profile known to have detrimental health impacts (25).

There has been relatively little work performed to describe the dietary intake and nutritional composition of the diets of African communities in the United Kingdom or the effects and extent of dietary acculturation. Historical reports have described the traditional West African diet as high carbohydrate, low total and saturated fat (26) which is believed to, at least in part, contribute to the cardioprotective lipid profile (27) and low rates of ischaemic heart disease (IHD) in these communities (28). In the present study, we have found carbohydrate remains the principle source of energy in the diet of both native and migrant Ghanaian adults but that UK-Ghanaians have a lower intake of carbohydrate partnered with a higher intake of fat, saturated fat, and protein compared with their native counterparts. Although the energy derived from saturated fat for both cohorts is within published guidelines for chronic disease prevention (23, 29) and supports recent evidence from the CHASE child cohort suggesting that the UK Black African diet has a cardio-protective fat profile (30), it is concerning to see the significantly higher intakes of saturated fat in the UK-Ghanaian diet compared to the native diet. The nutritional pattern of higher fat, saturated fat, and protein is particularly concerning given the recent analysis by Anderson et al. (12) who demonstrated in adults of West African origin that for every 1% increment in energy from protein, total fat, or saturated fat the risk of developing T2D increased by 9, 5, and 16%, respectively.

People of African ancestry are disproportionately affected by T2D, CVD, and other diet-related NCDs and it is recognised that these are more prevalent in West African communities following migration to ‘the West’ (11–13). The detrimental effects of an ‘urbanised’ lifestyle are recognised in driving the burden of chronic diseases in both native and diasporic communities and are therefore a target for both public health and healthcare management programmes (7, 31). The recent Benin longitudinal study has recognised the increased cardio-metabolic risk associated with a low diet quality score (based on frequency of vegetable, egg, meat, and dairy intake) in a West African population undergoing nutrition transition (31). Additionally, a number of recent epidemiological studies of West African populations have used dietary pattern analysis to identify the food patterns associated with increased NCD risk (9, 10, 32); however, such studies have not yet been performed in diasporic West African communities.

Explicit inequalities in healthcare provision are recognised to have impact on minority ethnic groups in the United Kingdom and elsewhere and a need to improve the cultural sensitivity of healthcare as well as developing targeted prevention strategies is recognised (3). The present study provides a detailed analysis of the foods that are important in the diets of people of Ghanaian ancestry in the United Kingdom, which is important for better informing healthcare practitioners who work with these communities. We have shown the importance of traditional foods and dishes as well as the role of ‘Western’ food items and how these contribute to the nutritional composition of the diet. This is the first report to describe important foods consumed in the diet of both UK-Ghanaians and native Ghanaian adults and to demonstrate the contribution they make to the nutritional profile of the diet, for example traditional ‘one-pot’ stews and soups and traditional starches such as yam, cassava, and fufu. Our findings draw agreement with those of the Australian GHANASIA study, which demonstrated a reduction in the intakes of traditional food sources (e.g. fufu as a principle source of carbohydrate and fish as a principle source of protein) following migration from Ghana to Australia (14), although it is evident that traditional dishes (e.g. soups and stews) are still of great importance in the diet, which agrees with previous reports (33, 34). Our data also demonstrates which non-traditional foods and dishes are prevalent in the diet of migrant Ghanaian adults, for example confectionery, processed meats and breakfast cereals, which explain some of the differences in nutritional composition that were seen, for example consumption of breakfast cereals and wholemeal bread contributing to the significantly higher NSP intake in the diet of UK-Ghanaians. It is clearly of benefit for healthcare practitioners to have a thorough knowledge of the food intakes and practices of important migrant groups. However, research has reported inadequate knowledge of traditional ethnic foods amongst nutritional practitioners (35). Therefore, the identification of TGFs and their contribution to nutrient intake as presented here allows a practical application to clinical and public health nutritional practice. Awareness of the types of foods in the traditional Ghanaian diet that are likely to contribute to a positive dietary fat profile (e.g. fish, and vegetable-based soups and stews) is important, as encouragement of these foods should inform culturally specific dietary advice. This supports previous observational research that highlighted the positive benefits of the native West African diet post-migration to Europe and Canada (36).

The strengths and limitations of the study warrant consideration. The majority of available literature on diet in diasporic African communities focuses on populations of Caribbean ancestry or uses broad classifications of ethnicity such as ‘African-American’ and ‘African-Caribbean’; in using these collective terms these studies describe a heterogenous population and fail to recognise distinct cultural practices. In our study, we have described a specific ethnic group and as a result have presented data that is of direct relevance to healthcare practitioners who care for members of the UK-Ghanaian communities and for the development of culturally-sensitive public health strategies. Our cohorts had similar characteristics, both were urban-dwelling with similar employment rates and similar body weights and BMIs; all of these factors are important confounders in dietary research. The dietary data were collected using detailed 3-day dietary records and ethnically sensitive photo aids for estimation of portion sizes; extensive information was gathered on traditional foods, recipes, and portion sizes while most of the studies in the literature have used a single 24-h recall (37, 38) or Food Frequency Questionnaires (FFQs) (12, 39–41) that are unlikely to have captured such detail. TGFs, dishes, and recipes were analysed as new entries in our nutritional composition database as the original database contains very few traditional African foods. We entered more than 300 new recipes or food stuffs into the database and are confident that these had a significant impact on our findings. In our study, we estimated that under-reporting may have been present in approximately 28% of the native and 62% of the migrant participants; the latter is higher than the general average of 30% (42) but not markedly different from Mennen et al.'s (40) study of migrant and traditional African-Caribbean communities which showed that under-reporting is higher in migrant communities. We appreciate that our participants are not representative of the wider UK West African communities; however, we believe our study is the biggest study to date to collect detailed food group and nutrient data through dietary records as opposed to FFQs. Furthermore, we are aware of other limitations: our participants were recruited predominantly from London and may not therefore be nationally representative; however, it is recognised from national records that London is the most important region in the United Kingdom with up to 80% of the African community residing within it (43). Our sample size was not sufficient to explore gender differences within and between ethnic groups, which may be an important line of investigation in future work (44). All dietary assessment methodologies have limitations associated with mis-reporting and misinterpretation; however, we are confident that our data has not been more greatly impacted by these limitations than other studies of this type; our data is comparable to other studies (45, 46). In analysing the nutritional composition of the diets of these communities, we are limited to reporting the principal macronutrients and fibre for which we had complete data. A notable limitation of our study is the lack of information on sodium intake. Breakfast cereal and processed meat are sources of dietary sodium, and the increased/novel consumption of these foods is of particular concern for the Black African population in the United Kingdom due to increased hypertension risk (2). The inherent weakness in the assessment of food records as a determinant of sodium intake is well documented (47), and although sodium intake was calculated by the current researchers from the dietary records, preliminary analysis did not produce scientifically robust data. This methodological limitation should be an important consideration for future researchers, where urinary sodium should be employed to validate intake. We appreciate the multidimensional nature of dietary acculturation (48); our quantitative methods did not enable us to explore the complexity of this phenomenon in our communities and further investigation of the reasons, attitudes and perceptions underlying these dietary practices would greatly assist public health and healthcare practitioners in working with these communities to instigate positive dietary practices. Finally, we did not collect data on any biomedical markers of health and are not able to draw any firm conclusions on the effects of our findings on the health of our participants. It is important that further studies are performed to understand the impact of dietary acculturation on chronic disease risk in these communities.

## Conclusion

In this study, we have presented novel data which demonstrates specific dietary differences between West African adults residing in Ghana compared to a first-generation migrant population residing in London. Our findings show that the migrant diet is characterised by significantly higher intakes of fat, saturated fat, and protein and significantly lower intakes of carbohydrate; a macronutrient profile believed to at least in part promote the increased risk of NCDs that is evident in this important minority ethnic group in the United Kingdom. These differences in the nutrient profile are likely to be modulated by the consumption of ‘Western’ foods observed in migrant communities. Therefore, encouraging traditional food consumption in the UK-Ghanaian population may be a beneficial nutritional intervention. The results presented here may assist nutritional practitioners in developing a culturally competent knowledge base to address health inequalities.
